# Production of Nanocellulose from Sugarcane Bagasse and Development of Nanocellulose Conjugated with Polylysine for Fumonisin B1 Toxicity Absorption

**DOI:** 10.3390/polym16131881

**Published:** 2024-07-01

**Authors:** Parichat Thipchai, Korawan Sringarm, Winita Punyodom, Kittisak Jantanasakulwong, Sarinthip Thanakkasaranee, Rangsan Panyathip, Chaiwat Arjin, Pornchai Rachtanapun

**Affiliations:** 1 Doctor of Philosophy Program in Nanoscience and Nanotechnology (International Program/Interdisciplinary), Faculty of Science, Chiang Mai University, Chiang Mai 50200, Thailand; parichat.t245@gmail.com; 2 Department of Animal and Aquatic Sciences, Faculty of Agriculture, Chiang Mai University, Chiang Mai 50200, Thailand; korawan.s@cmu.ac.th (K.S.); chaiwat.arjin@cmu.ac.th (C.A.); 3 Center of Excellence in Agro Bio-Circular-Green Industry (Agro BCG), Chiang Mai University, Chiang Mai 50200, Thailand; jantanasakulwong.k@gmail.com (K.J.); sarinthip.t@cmu.ac.th (S.T.); 4 Department of Chemistry, Faculty of Science, Chiang Mai University, Chiang Mai 50200, Thailand; winitacmu@gmail.com; 5 Center of Excellence in Materials Science and Technology, Chiang Mai University, Chiang Mai 50200, Thailand; 6 Division of Packaging Technology, School of Agro-Industry, Faculty of Agro-Industry, Chiang Mai University, Chiang Mai 50100, Thailand; rangsanpanyatip@gmail.com; 7 Office of Research Administration, Chiang Mai University, Chiang Mai 50200, Thailand

**Keywords:** nanocellulose, sugarcane bagasse, polylysine, adsorption, fumonisin B1

## Abstract

The present study aimed to extract nanocellulose (NC) from sugarcane bagasse agricultural waste through a chemical method (sulfuric acid hydrolysis and ultrasonication). Subsequently, the nanocellulose product was conjugated with polylysine (NC–PL) and assessed for its efficacy in reducing the toxicity of Fumonisin B1 (FB1), a mycotoxin produced by fungi commonly found in corn, wheat, and other grains. Experimental results confirmed the successful conjugation of NC and PL, as evidenced by FTIR peaks at 1635 and 1625 cm^−1^ indicating amide I and amide II vibrations in polylysine (PL). SEM analysis revealed a larger size due to PL coating, consistent with DLS results showing the increased size and positive charge (38.0 mV) on the NC–PL surface. Moreover, the effect of FB1 adsorption by NC and NC–PL was evaluated at various concentrations (0–200,000 μg/mL). NC–PL demonstrated the ability to adsorb FB1 at concentrations of 2000, 20,000, and 200,000 μg/mL, with adsorption efficiencies of 94.4–100%. Human hepatocellular carcinoma (HepG2) cells were utilized to assess NC and NC–PL cytotoxic effects. This result is a preliminary step towards standardizing results for future studies on their application as novel FB1 binders in food, food packaging, and functional feeds.

## 1. Introduction

Nowadays, the phenomenon of global climate warming has triggered growing apprehensions regarding the state of the global environment. As a result, there has been a notable upsurge in interest surrounding the utilization of lignocellulosic biomass as a substitute for materials sourced from fossil fuels [1]. The conversion of lignocellulosic biomass into valuable products not only reduces waste but also fosters sustainable development across various industries [2]. In particular, a large amount of sugarcane bagasse is left over in the sugar production industry. This excess is generally removed by combustion, leading to environmental concerns such as air pollution and health risks. Eliminating this biomass is a major challenge in many countries worldwide [3,4]. However, bagasse is considered a valuable material due to its high cellulose content, which ranges from 40–50% [5,6]. This cellulose can be extracted and used for various purposes, including chemical production, energy generation, and material development [5]. Using cellulose from sugarcane bagasse not only enhances the worth of this agricultural excess but also mitigates environmental repercussions by diverting it from incineration.

Cellulose is a significant structure of all plant materials that gives plant cell walls strength and is composed of homopolysaccharide linear chains formed of β-d-glucopyranose units linked together by β-1-4-glycosidic bonds. Its main components are carbon, hydrogen, and oxygen [7,8]. Cellulose is a renewable resource and eco-friendly biodegradable material used widely in textiles, paper, and food products [9]. Nanocellulose boasts remarkable properties due to its nano-sized fibers. Nanocellulose has received a lot of attention among researchers because nanocellulose has excellent physical properties and yields high-value products. Furthermore, nanocellulose has small dimensions, a variety of shapes, a high surface area, a large capacity to hold water, and a highly reactive surface of the –OH side group [10]. In addition, nanocellulose has low environmental effects and has potential in the fields of pharmaceuticals [11,12], food [13], packaging [14], and other industries [15]. Recent research has explored its potential in various applications, particularly in the development of biocompatible materials for toxin removal. In the realm of environmental remediation, nanocellulose-based composites have been designed to remove contaminants from wastewater [16]. Furthermore, nanocellulose has emerged as a valuable component in combination with other biopolymers aimed at addressing natural toxins contamination [17].

Mycotoxin contamination in crops and agricultural products poses significant economic and health risks such as reduced yields, increased costs, and potential chronic illnesses, including cancer, in humans and animals [18]. Fumonisin B1 (FB1), produced mainly by the fungus *Fusarium verticillioides*, is a significant mycotoxin contaminating various foodstuffs globally, such as corn and other grains. It causes food spoilage and poses serious health risks, with toxic effects on the liver, brain, and kidneys [19]. FB1 is generally found in food processing and is hard to eliminate from food due to its heat and pressure resistance. Thus, the Good Agricultural Practices (GAP) standard was performed to manage contamination, and to control and limit the detoxification methods, including biological, chemical, and physical approaches. However, the many previous methods often have side effects and drawbacks for food quality and safety [20,21]. Consequently, nanocellulose with biocompatibility was investigated and examined in previous studies. For example, nanocellulose was integrated with retinoic acid, using mycotoxin adsorption to maintain food quality without harmful residues [22,23,24]. Then, the modified nanocellulose with polylysine (PL) was applied for antibacterial activity, showing a high antifungal activity [25]. For the food industry, PL is also provided as the food preservation agent, improving the tissue culture in cell adhesion [26]. For other innovations, the integrated PL with nanocellulose was designed for a biodegradable aerogel between TEMPO-oxidized nanocellulose and polylysine with a citric acid cross-linking agent, demonstrating the soil degradation of food packaging, and helping to obtain a hemolysis rate under 5% in medical research [27]. Similarly, nanocellulose and polylysine-based on 3D composite scaffolds were studied for wound healing applications [28]. However, the nanocellulose innovation of using sugarcane bagasse fibers, a waste product of the sugar industry, for FB1 adsorption has been a rare topic of study [29].

This work aims to study the extracted nanocellulose from sugarcane bagasse waste using a chemical and mechanical method involving sulfuric acid hydrolysis and ultrasonication. Additionally, the extracted nanocellulose was modified with polylysine through a conjugation process. The chemical composition and yield of raw fiber (RF), cellulose (CL), and nanocellulose (NC) were analyzed. The size and morphology of raw fiber (RF), cellulose (CL), nanocellulose (NC), carboxy-nanocellulose (C–NC), and polylysine conjugated nanocellulose (NC–PL) derived from sugarcane bagasse were characterized using dynamic light scattering (DLS) and scanning electron microscopy (SEM). All chemical structures of the provided cellulose and nanocellulose were examined with Fourier transform infrared (FTIR) spectroscopy. In particular, the NC and NC–PL were evaluated for the potential to adsorb FB1 at various concentrations (0–200,000 μg/mL) using a competitive enzyme-linked immunosorbent assay (ELISA). Significantly, MTT assays were used to test the preliminary cytotoxicity of human hepatocellular carcinoma (HepG2) cells after exposure to NC and NC–PL. 

## 2. Materials and Methods

### 2.1. Materials 

Acetic acid glacial, sodium hydroxide, sodium chlorite, citric acid, dimethyl sulfoxide (DMSO), and sulfuric acid, all of the analytical reagent (AR) grade, were procured from RCI Labscan Ltd. (Bangkok, Thailand). Sugarcane bagasse (*Saccharum officinarum* L.) was sourced from our previse work [30]. Additionally, poly-L-lysine hydrobromide (30–70 kDa; P2636), 1-ethyl-3-(3-dimethylaminopropyl) carbodiimide (EDC), FB1, and 3-(4,5-dimethyl)-2,5-diphenyltetrazolium bromide (MTT) were supplied by Sigma–Aldrich Company (St. Louis, MO, USA). Dulbecco’s modified eagle medium (DMEM) medium was obtained from Invitrogen, Waltham, MA, USA.

### 2.2. Extraction of NC and Conjugation with PL

Sulfuric acid hydrolysis was utilized to extract NC, following our prior research [30]. Briefly, the fibers were extracted through an alkaline treatment to remove hemicellulose and other substances. Following drying, the sample underwent sodium chlorite bleaching to eliminate residual lignin to obtain CL. Subsequently, CL (10 g) was hydrolyzed with 32% *v*/*v* sulfuric acid for 5 h at 50 °C with continuous stirring using a magnetic stirrer hot plate (IKA^®^ C-MAG HS 7, Wilmington, NC, USA). The resulting hydrolyzed CL was centrifuged (Centrifuge, BKC-TL511, Jinan Biobase Biotech, Co., Ltd., Jinan, China) at 4500 rpm for 10 min. Sodium hydroxide (5% *w*/*v*) was added until the solution reached pH 7. The NC was then sonicated in an ice bath for 30 min using an ultrasonic generator (Ultrasonic Homogenizer, BEM-900A, Bueno-Biotech Co., Ltd., Nanjing, China). Finally, the sonicated NC was centrifuged at 5000 rpm for 10 min until no precipitation occurred. To initiate conjugation, 20 mL of NC at a concentration of 1 g/mL was mixed with 20 mL of 5% (*w*/*w*) citric acid and heated to 100 °C for 15 min. The C–NC was washed with distilled water through centrifugation at 6000 rpm for 20 min. Subsequently, 100 mg of C–NC was conjugated with 1 mL of 100 mg/mL PL and 0.1 mL of 100 mg/mL EDC. The mixture was then incubated for 30 min at 37 °C. Finally, the NC–PL was washed with distilled water following centrifugation at 12,000 rpm for 15 min. The yield was determined by dividing the freeze-dried (DW-10N Freeze Dryer, Chongqing Drawell Instrument Co., Ltd., Chongqing, China) weight of NC obtained after acid hydrolysis by the initial freeze-dried and weight of cellulose. Finally, the NC, C–NC, and NC–PL suspensions were freeze-dried for characterization purposes [14]. 

### 2.3. Chemical Composition and Yield

The chemical composition analysis of RF, CL, and NC followed our previous methods [14]. In brief, lignin content in 1 g samples was determined through hydrolysis in 15 mL of 72% sulfuric acid, followed by dilution to 3% sulfuric acid concentration over 4 h. Reflux condenser operation overnight ensured volume stability. Lignin was filtered, washed, and dried, and its content was computed per the Technical Association of the Pulp and Paper Industry (TAPPI) standard [31]. The α-cellulose and hemicellulose were extracted without extractives using the Browning technique (Browning, The Chemistry of Wood, Wiley, New York, NY, USA, 1963) and separated via 17.5% *w*/*v* sodium hydroxide at 25 ± 0.2 °C, following the TAPPI standard [32] for sample evaluation. Equation (1) was utilized to get the percentage yield of the sample products.

(1) $$\mathrm{Yield}\;\mathrm{of}\;\mathrm{cellulose}\;\left(\%\right)=\frac{Wa}{Wb\;}\times 100$$

where *Wa* and *Wb* were the weights of sample products after and before extraction, respectively.

### 2.4. Fourier Transform Infrared Spectroscopy (FTIR)

The FTIR spectrometers (FT-IR Spectrometer, FT/IR-4700, JASCO International Co., Ltd., Pfungstadt, Germany) were employed to analyze the functional groups of RF, CL, NC, C–NC, and NC–PL. Approximately 2 mg of samples were pulverized and mixed with KBr. These mixtures were then formed into pellets and inserted into the apparatus. Transmission measurements were conducted in the wavenumber range of 4000–500 cm^−1^ using a scan rate of 64, following the methodology established in our previous study [30].

### 2.5. The Morphological Analysis

The morphological analysis of RF and CL was conducted using a scanning electron microscope (SEM, JSM-IT300, JEOL Ltd., Peabody, MA, USA), following our previous research [30]. Samples were prepared by affixing them to a carbon tab on a brass stub and coating them with gold for 30 s. The SEM examination was conducted at 500× magnification and 15 kV voltage. For the analysis of NC, C–NC, and NC–PL, a field emission scanning electron microscope (FE-SEM) with STEM capability (FE-SEM, JSM-IT800, JEOL Ltd., Peabody, MA, USA) was utilized. Samples were dispersed in distilled water to achieve a concentration of 0.01%, followed by deposition onto a Cu grid and exposure to light for 15 min for drying. Observation was conducted at magnifications ranging from 10,000× to 30,000× and a voltage of 20 kV.

### 2.6. Measuring the Particle Size by Dynamic Light Scattering (DLS) and Zeta Potential Measurements

The particle sizes and zeta potential of NC, C–NC, and NC–PL suspensions were measured by Zetasizer (Malvern, Worcestershire, UK). The average particle size was calculated using DTS software version 5.00 (Malvern, Worcestershire, UK), following our earlier study. Each test was run three times for every sample [30]. 

### 2.7. Adsorption of FB1 by NC and NC–PL 

Various conditions, including different concentrations of NC and NC–PL, were investigated. In this study, 100 μL of NC and NC–PL at concentrations of 0, 20, 200, 2000, 20,000, and 200,000 μg/mL were added to 100 μL of 100 ng/mL FB1, followed by mixing using a Vortex Mixer (Vortex-Genie^®^ 2; Scientific Industries, Bohemia, NY, USA) and incubation at 37 °C for 3 h. After incubation, all tubes were centrifuged at 12,000 rpm for 10 min, and the optical density (OD) of the supernatant was measured. The determination and quantification of total FB1 content were conducted using a competitive enzyme-linked immunosorbent assay ELISA (RIDASCREEN^®^FAST Fumonisin ECO Art. No. R5603 kit from r-Biopharm, Darmstadt, Germany), and measured by a fluorescence microplate reader (Biotek, Winooski, VT, USA) at 450 nm. Then, the percentage of adsorption was calculated using the adsorption equation formula described by Pan et al. [33].

### 2.8. Cell Culture 

Human hepatocellular carcinoma (HepG2) cells (HB-8065, American Tissue Collection Center (ATCC), Manassas, VA, USA) were grown in DMEM medium containing 10% fetal bovine serum (FBS) and 1% penicillin–streptomycin. The cell lines were cultured in a humidified incubator at 37 °C with 5% CO_2_. 

### 2.9. The Cytotoxicity of NC and NC–PL by MTT Assay

HepG2 cells (1 × 10^6^) were cultured and treated with NC and NC–PL at 0, 20, 200, 2000, and 20,000 μg/mL concentrations for 72 h at 37 °C in a 5% CO_2_ atmosphere. Cell viability was assessed using the MTT assay [34]. Briefly, MTT solution (5 mg/mL) was added to the cell media to achieve a final concentration of 500 μg/mL, and the samples were incubated for 4 h at 37 °C in a 5% CO_2_ atmosphere. The medium was then removed, and the cells were treated with DMSO for 30 min. The absorbance of the cells was measured at 580 nm and 660 nm using a microtiter plate reader (Biotek, Winooski, VT, USA). The percentage of cell viability was calculated according to Equation (2) [35].

(2) $$\mathrm{Cell}\;\mathrm{viability}\;\left(\%\right)=\frac{A}{B\;}\times 100$$

where *A* represents the optical density (OD) of the sample well, and *B* represents the OD of the negative control, the cell suspension not exposed to NC and NC–PL is considered the negative control.

### 2.10. Statistical Analysis

Averages and standard deviations have been calculated for every data set. At a significant threshold of *p*-value 0.05, one-way ANOVA was used to calculate the significance of differences. SPSS Statistics (IBM SPSS Statistics 27.0, New York, NY, USA) was used for statistical analysis.

## 3. Results and Discussion

### 3.1. Chemical Composition and Yield 

**Figure 1:**
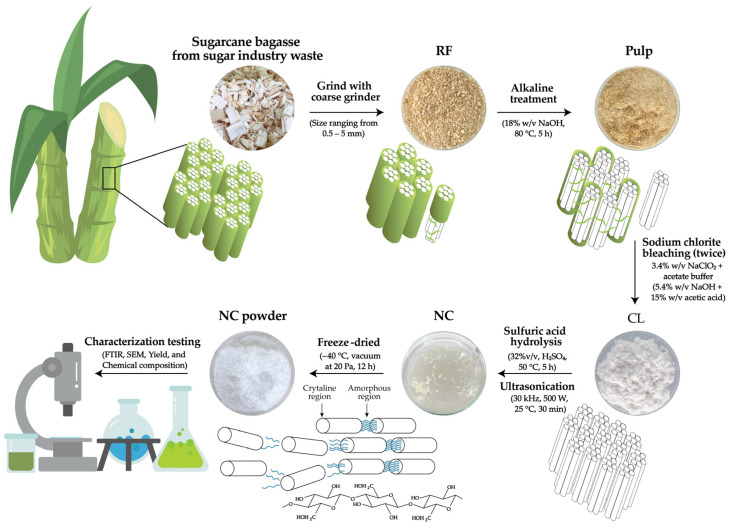
Schematics of CL and NC production from sugarcane bagasse fiber extraction.

The objective of this study was to isolate NC from sugarcane bagasse waste and subsequently modify it to NC–PL. Initially, CL was isolated through a series of treatments including alkaline and bleaching procedures, followed by hydrolysis with sulfuric acid to obtain NC. The CL and NC yield was determined gravimetrically, calculated as the percentage of dried NC over the initial dry weight of sugarcane bagasse. This was achieved by comparing the final freeze-dried weight of NC obtained after acid hydrolysis to the initial freeze-dried weight of cellulose. The resulting CL and NC yield was 52.3% and 32.4%, respectively (Table 1). The observed values were higher than those reported in previous research, which found that NC yields from sugarcane bagasse were 27.7% [4]. Moreover, the results are consistent with previous research from Nang et al. [36] that reported NC yields from various biomass sources, with yields ranging from approximately 28.6–40.1%. The yield of CL decreases after extraction due to the removal of non-cellulosic components (hemicellulose, lignin), which reduces the yield of CL in Figure 1. The cellulose content from sugarcane bagasse corresponded with the previous result (40–55%) [37]. In addition, the sulfuric acid hydrolysis extraction process selectively breaks down the amorphous regions of cellulose while preserving the crystalline regions, thus reducing the yield of NC (Figure 1). In addition to the types of raw materials, various factors lead to different yields of NC. These factors include the hydrolysis duration, starting material quality, ultrasonication effectiveness, purification losses, and reaction conditions (such as temperature and pH) [38].

The chemical compositions of RF, CL, and NC of sugarcane bagasse were compared, as presented in Table 1. The α-cellulose composition of CL and NC increased from 56% to 87.3%, compared to 41.7% in RF from sugarcane bagasse fibers. These results of sugarcane bagasse data align well with previous reports, as indicated in Table 1. The increase in α-cellulose in NC leads to a rise in its crystallinity, as shown in Figure 1, a schematic of cellulose and NC production. Lignin and hemicellulose percentages in CL decrease after sodium chlorite bleaching because the sodium chlorite acts as an oxidizing agent, breaking down these components into smaller, soluble molecules that can be easily washed away, thereby increasing the purity of the cellulose [14]. In the context of NC, lignin and hemicellulose have lower percentages compared to CL because sulfuric acid selectively hydrolyzes the amorphous regions of cellulose, breaking down the hemicellulose and lignin while maintaining the crystalline regions of cellulose. NC has several advantages over bulk cellulose, including higher strength and stiffness, a larger surface area, transparency, excellent barrier properties, lightweight nature, versatility for chemical modification, and improved rheological properties [39]. The above results indicate that NC has been recognized as one of the most exceptional renewable materials in biomass.

**Table 1:** Chemical composition and yield of RF, CL, and NC from sugarcane bagasse.

Samples	α-Cellulose (%)	Hemicellulose (%)	Lignin (%)	Yield (%)	References
RF	41.4–58.8	20–32	17–32	–	[5,6,40]
CL	56–42	12.5–34	18–22	40–55	[37,39,41]
NC	87.3–81.0	6.9–12	2.5–6.5	27.7–40.1	[3,4,36,41]
RF	41.7 ± 1.3 ^c^	29.1 ± 1.1 ^a^	19.7 ± 0.5 ^a^	–	Our work
CL	68.3 ± 1.4 ^b^	6.5 ± 0.8 ^b^	3.7 ± 0.5 ^b^	52.3 ± 1.5 ^a^	Our work
NC	90.1 ± 1.3 ^a^	3.5 ± 0.6 ^c^	0.6 ± 0.3 ^c^	32.4 ± 0.2 ^b^	Our work

Mean ± standard deviation values within a column in the same group; those followed by different letters (a–c) are significantly different at *p* < 0.05.

### 3.2. Fourier Transform Infrared Spectroscopy (FTIR) 

**Figure 2:**
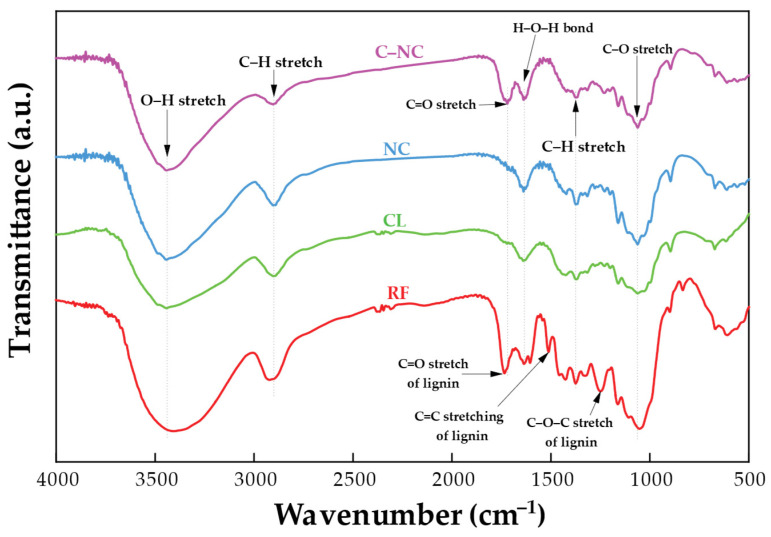
FTIR spectrum of RF, CL, NC, and C–NC.

As a result, FTIR analysis was conducted to assess the chemical structure changes in the initial samples RF, CL, NC, and C–NC, as depicted in Figure 2 and Table 2. The dotted lines delineate changes before and after chemical extraction. All samples exhibit different regions, characterized by two main absorption regions: one in the high wavenumber region (2800–3500 cm^−1^) and one in the low wavenumber region (700–1750 cm^−1^). The peak centered at 2900 cm^−1^ corresponds to the stretching vibrations of C–H groups in cellulose [42], while the broad peak around 3445 cm^−1^ arises from O–H stretching vibrations of hydroxyl groups within cellulose molecules [43]. However, alkali treatment and bleaching induced structural changes in the samples, as evident in the FTIR spectra. In the RF spectrum, the peak around 1734 cm^−1^ corresponds to C=O stretching vibrations, originating from hemicellulose or lignin components, which are absent in spectra post-alkaline and bleaching treatments due to the removal of these components [44]. Furthermore, absorbance peaks at 1605, 1510, 1240, 830, and 780 cm^−1^, attributed to the elongation of aromatic rings in lignin, disappear after such treatments [45,46,47,48,49]. The peak at 1635 cm^−1^ observed in all samples is attributed to the H–O–H stretching vibrations of water [7]. The β-glucosidic linkage between sugar units is reflected by a peak at 895 cm^−1^, while the elongation of hemicellulose in lignin is indicated by a peak at 1060 cm^−1^, demonstrating the preservation of cellulose molecular structure characteristics [14,50]. During hydrolysis, the increase in the CL peak intensity suggests a decrease in other components. The slight increase in the intensity of small narrow peaks at 3490, 3445, and 1060 cm^−1^ in the NC peak could be attributed to hydrolysis [51,52]. The C–NC peak at 1720 cm^−1^ significantly increased after reacting with citric acid, following the C=C stretching [53,54].

**Figure 3:**
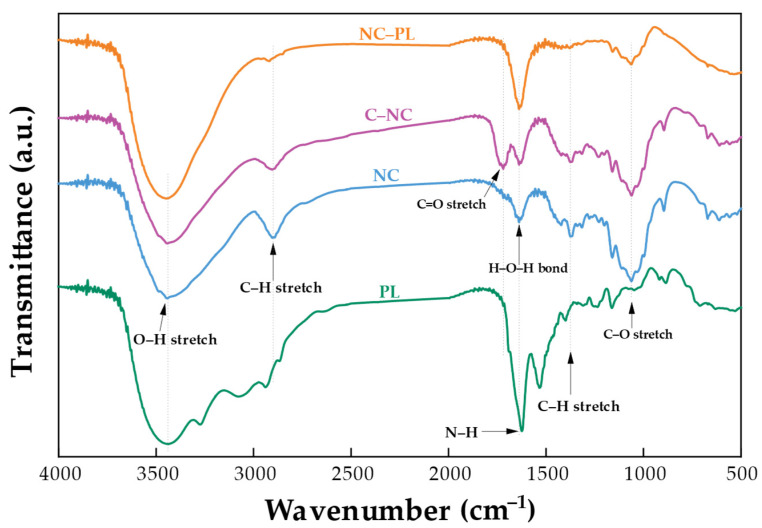
FTIR spectrum comparison of PL, NC, C–NC, and NC–PL.

FTIR test results depicted in Figure 3 and Table 2 compare the chemical structure changes in PL, NC, C–NC, and NC–PL. The FTIR spectrum of NC–PL revealed the disappearance of peaks centered at 3270, 3070, 2940, 2866, 1625, 1530, and 1160 cm^−1^ from the PL spectrum after the reaction with C–NC [55]. The C–NC peak at 1720 cm^−1^ also disappeared, confirming binding between NC and PL structures. The peak at 3448 cm^−1^ of NC–PL exhibited higher intensity than NC and C–NC due to the overlap between primary and tertiary amine N–H stretching vibration and O–H stretching vibration. Additionally, peaks at 1635 and 1625 cm^−1^ in NC–PL showed a noticeable increase in intensity, attributed to amide I and amide II vibrations in PL [55,56]. In this study, NC was synthesized and then modified using citric acid (Figure S1a). Following this modification, the C–NC was conjugated with PL. The conjugation process involved the use of EDC, which acts as a coupling agent to facilitate the formation of covalent bonds between the carboxyl groups on the citric acid-modified NC and the amine groups present on PL. The reaction mechanism is illustrated in Figure S1b, which shows how EDC mediates the linkage between the carboxyl and amine groups, resulting in the conjugated NC–PL complex [57].

### 3.3. Morphological Analysis 

**Figure 4:**
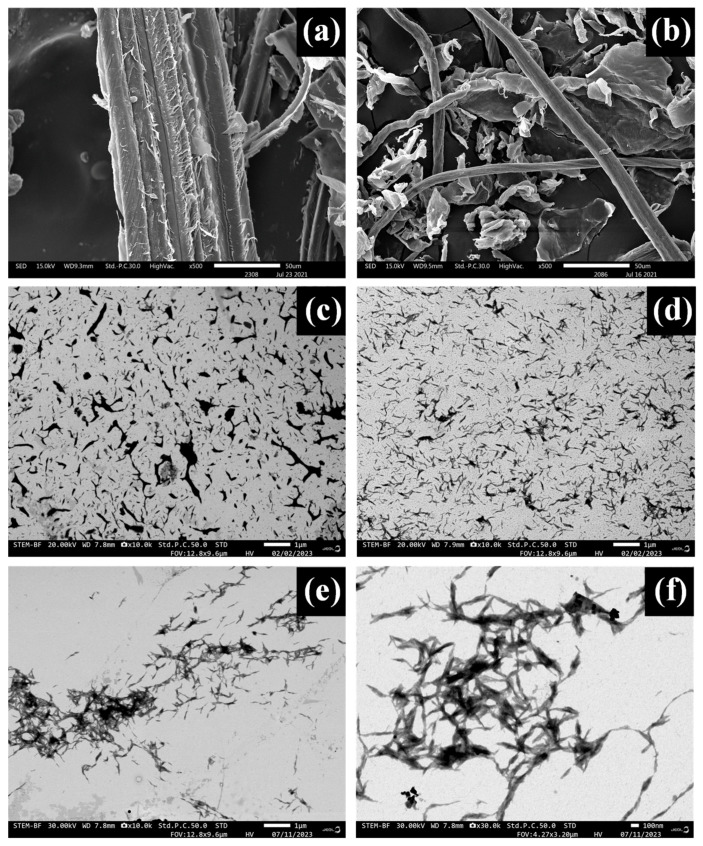
SEM of sugarcane bagasse: (**a**) RF, (**b**) CL, (**c**) NC, (**d**) C–NC, (**e**) NC–PL at 10,000×, and (**f**) NC–PL at 30,000×.

Figure 4a illustrates the morphology of RF extracted from sugarcane bagasse, showcasing an overall diameter of 92.1 × 10^3^ nm (Table 3). Sugarcane bagasse fibers primarily consist of parenchymatous ground tissue intertwined with vascular bundles composed mainly of cellulose, hemicellulose, and lignin. Cellulose provides structural strength, hemicellulose acts as a bonding agent, and lignin binds the fibers together. Additionally, various substances such as pectins and other polymers are also present within the fibers [58]. Figure 4b depicts CL fragments and fibers of 7.6 × 10^3^ nm in diameter post-bleaching and details are in Table 3. Treatment with NaOH and NaClO_2_ solutions leads to thinning and shortening of CL. SEM results show sugarcane bagasse-derived CL has a strand and lump-like morphology. 

Figure 4c–f presents the morphology analysis of NC, C–NC, and NC–PL, respectively. The particles in all samples exhibited a rod shape. SEM results reveal the correlation between the diameter and length of the samples as summarized in Table 3, with reported values for NC being 33.4 nm and 224.8 nm, for C–NC being 44.3 nm and 265.3 nm, and for NC–PL being 53.6 nm and 363.5 nm, respectively. Notably, the smallest diameter and length were observed in NC. Furthermore, agglomeration occurred as the pH was neutralized after the hydrolysis of sulfuric acid [59].

In Figure 4d, it was observed that C–NC treated with citric acid exhibited improved dispersion due to the lower pH value, attributed to a clear increase in C=C groups evident in FTIR spectra and confirmed experimentally during the reaction of C–NC with citric acid. Subsequently, citric acid was conjugated with the substance. Additionally, in Figure 4e,f, NC–PL exhibited a larger size attributed to its coating with PL [57]. Consequently, the average fiber diameter of NC–PL was higher compared to C–NC and NC, as indicated in Table 3; this observation aligns with previous FTIR results.

### 3.4. Measuring the Particle Size by Dynamic Light Scattering (DLS) and Zeta Potential Measurements

**Figure 5:**
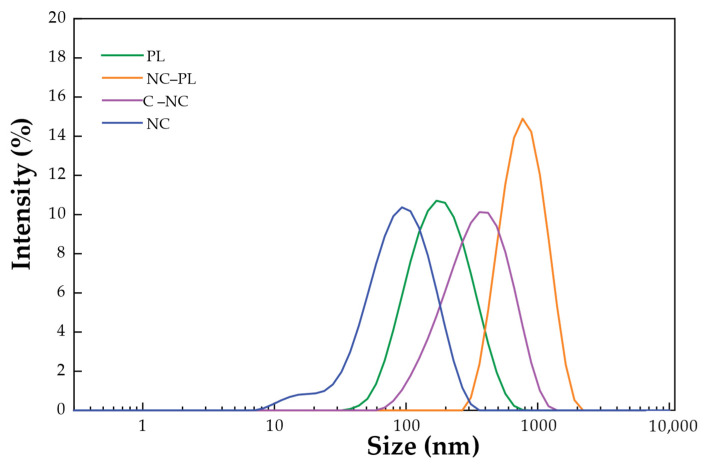
Particle size analysis from the DLS technique of PL, NC, C–NC, and NC–PL.

The particle sizes of PL, NC, C–NC, and NC–PL were analyzed using the DLS technique, and the results are summarized in Figure 5. The DLS measurements yielded particle size values of 174.1 nm for PL, 112.1 nm for NC, 439.0 nm for C–NC, and 867.0 nm for NC–PL, respectively. These results indicate a bimodal size distribution for the samples, which is consistent with the observations from the SEM image shown in Figure 4. Notably, NC–PL demonstrated the largest particle size among the samples analyzed, confirming the binding between NC and PL structures [53]. 

In Table 4, the zeta potential measurements of PL, NC, C–NC, and NC–PL are shown. The highest zeta potential (−64.7 mV) and the smallest average size of NC may be due to the stabilization of the NC sample by electrostatic repulsion [60]. This occurs after hydrolysis, which increases the surface density of dissociated sulfonic acid groups on the NC, as evidenced by the zeta potential [39]. NC was modified by adding citric acid into NC, resulting in the highly forming C–NC (−39.7 mV). By contrast, the lowest zeta potential of NC–PL (38.0 mV) corresponds to the largest average size due to the conjugated structural PL. The amino groups in the PL shell are responsible for providing positive charges as well as serving as anchoring groups [61]. The zeta potential relates to the stability of colloidal dispersions. Zeta potential measures the strength of repulsion or attraction between particles. Higher zeta potential values (negative) indicate smaller molecules or dispersed particles and a more stable system, resisting aggregation. Conversely, lower zeta potential values (positive) suggest a greater tendency for particles to coagulate or agglomerate, leading to the destabilization of the dispersion [60].

### 3.5. Adsorption of FB1 by NC and NC–PL

**Figure 6:**
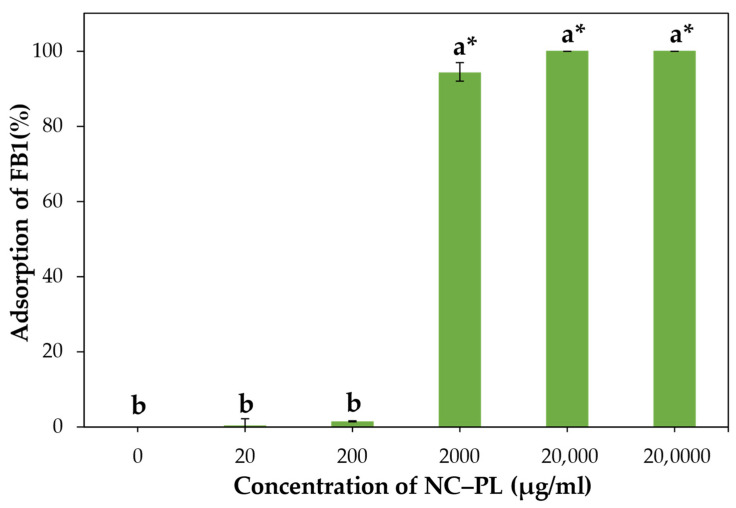
Adsorption of FB1 was evaluated after exposure to serial concentrations of NC–PL (0, 200, 2000, 20,000, and 200,000 μg/mL) for 3 h, prior to conducting the ELISA. The results are shown as mean ± standard deviation; a,b * (*p* < 0.05).

The toxicity of FB1, a mycotoxin produced by *Fusarium verticillioides*, is primarily attributed to its structure, which resembles that of sphingolipids. This resemblance enables FB1 to interfere with sphingolipid metabolism, crucial for cell membrane integrity and signaling. The critical part of the FB1 structure responsible for its toxic effects is the tricarballylic acid side chains attached to the C14 and C15 positions of the molecule. FB1 has a complex structure with several hydroxyl groups and a carboxyl group, making it negatively charged at physiological pH, as illustrated in Figure S2 [62,63]. This study investigated the absorption efficiency of NC–PL for FB1 under various concentrations of 0, 20, 200, 2000, 20,000, and 200,000 μg/mL, with an FB1 concentration of 100 ng/mL. In Figure 6, the percentage of FB1 adsorption revealed that concentrations of NC–PL at 2000, 20,000, and 200,000 μg/mL exhibited higher absorption rates (94.4%, 100%, and 100%, respectively) compared to concentrations of 0, 20, and 200 μg/mL, which showed 0%, 0.1%, and 1.5% absorption. Overall, these findings demonstrate the efficiency of NC–PL in absorbing FB1 toxins, particularly at higher concentrations. NC has a high surface area, making it an excellent material for adsorption purposes that can be chemically modified to improve its binding capacity by PL (Figure S1) which has multiple positive charges along its length. These positive charges (NH_3_^+^ ions) can interact with negatively charged molecules of the negatively charged (COO^−^ ions) FB1. Additionally, hydrogen bonding also plays a role in the binding process as shown in Figure S2 [63]. The research results are consistent with previous studies. Previous work demonstrated the binding of FB1, by commercial nanocellulose modified with polylysine [64]. However, only a few studies have been recorded in the database regarding the adsorption properties of NC–PL. Solis-Cruz et al. [65] compared the adsorption of chitosan with three cellulosic polymers on six mycotoxins. They found that cellulosic polymers could adsorb all of the mycotoxins, with an adsorption range of 34–52.9%. Notably, higher concentrations of FB1 led to increased adsorption. This significant finding underscores the importance of further investigating NC–PL extracted from sugarcane bagasse waste. Expanding this investigation to include a variety of agricultural sources can provide valuable insights into the versatility and potential applications of NC–PL.

### 3.6. The Effect of NC and NC–PL on HepG2 Cells

**Figure 7:**
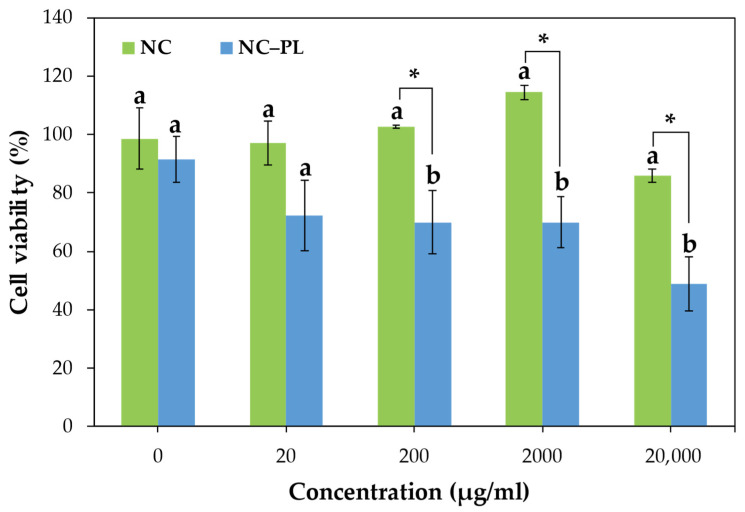
Cytotoxicity of NC and NC–PL on HepG2 cells was determined by the MTT assay. HepG2 cells were incubated with various concentrations (0, 20, 200, 2000, and 20,000 μg/mL) of NC and NC–PL for 72 h prior to the MTT assay. The results are shown as mean ± standard deviation and the values in the same group followed by a and b are significantly different (* *p* < 0.05).

The cytotoxicity of NC and NC–PL on HepG2 cells was determined by the MTT assay, as depicted in Figure 7. NC exhibited non-toxic characteristics toward HepG2 cells. Even at the high concentration of 20,000 μg/mL, NC exhibited non-toxic characteristics towards HepG2 cells, with a cell viability rate of 80–100%. This suggests that NC has potential for applications in various fields including medicine, pharmaceuticals, food packaging, food, and functional feeds, as it demonstrates non-toxicity to cells [66]. On the other hand, NC–PL can lead to cytotoxic effects, particularly at high concentrations (20, 200, 2000, and 20,000 μg/mL), with a cell viability of 72.3%, 70.0%, 69.9%, and 48.9%, respectively. Previous research on the cytotoxic effects of PL is influenced by several important factors, including the number of amino acids in PL and the type of target cell [67]. Meanwhile, PL is a cationic macromolecule that can interact with cells electrostatically and aids intracellular delivery [68]. However, NC–PL at a concentration of 2000 and 20,000 μg/mL can adsorb FB1 (Figure 6) while maintaining cell viability. This cytotoxicity was still at an experimentally acceptable level, with a viability rate of more than 50%, indicating that at least half of the cells survived the exposure to NC–PL. This suggests that the toxicity was not severe enough to significantly impair cell function or survival [69]. The findings suggest that this concentration may be deemed suitable for applications targeting the absorption of FB1 toxicity while ensuring the preservation of HepG2 cell viability. In terms of food and feed applications, NC–PL can be used as novel food preservatives or in the packaging of various foods. These applications deserve more research in future studies. 

## 4. Conclusions

This study utilized sugarcane bagasse, an agricultural waste from the sugar and alcohol industries in Thailand, as the raw material for extracting NC and modifying it to NC–PL. The successful preparation of NC–PL was achieved, showcasing its efficacy in absorbing mycotoxins. These results highlight the potential of NC–PL as a functional material for mycotoxin mitigation in various industries. In terms of food and feed applications, NC–PL can be utilized as novel food preservatives or integrated into the packaging of various food items. These potential applications warrant further exploration in future studies.

## Figures and Tables

**Table 2:** Assignments of FTIR peaks for Figure 2 and Figure 3 delineate the distinctive bonding in samples.

Frequency (cm^−1^)						Functional Group	References
RF	CL	NC	C–NC	PL	NC–PL		
3400	3445	3445	3445	−	−	O–H stretching vibration	[43]
−	−	−	−	3448, 3270, 3070	3448	Primary and tertiary amine N–H stretching vibration	[55,56]
−	−	−	−	2940, 2866	2920, 2855	–CH_2_ stretching vibration	[55,56]
2920	2900	2900	2900	−	−	C–H stretching of cellulose	[42]
1734	−	−	−	−	−	C=O stretching	[44]
−	−	−	1720	−	−	C=O stretching	[53,54]
1635	1635	1635	1635	−	1635	H–O–H bond of lignin	[7]
1605	−	−	−	−	−	Aromatic rings from lignin alcohol	[45]
−	−	−	−	1625, 1530	1630	Amide I and amide II vibrations	[55,56]
1510	−	−	−	−	−	Benzene ring C=C vibration in lignin.	[46]
1428	1428	1428	1428	−	−	Nearly aromatic C–H in-plane stretching	[47]
1374	1374	1374	1374	−	−	Phenolic O–H and C–H in methyl groups	[47]
−	−	−	−	1257, 1160	−	C–N stretching vibration	[55,56]
1240	−	−	−	−	−	C–O–C stretching	[47,48]
1159	1159	1159	1159	−	1159	C–H of cuaiacyl and syringyl	[47]
1110	1110, 1032	1110, 1032	1110, 1032	−	−	Syringyl ring aromatic C–H in-plane deformation	[51]
1060	1060	1060	1060	−	1060	Cellulose C–OH antisymmetric bend	[51,52]
−	995	995	995	−	−	Cellulose –CH_2_ stretching	[46]
895	895	895	895	−	−	Glycosidic linkage –C–H vibration	[14,50]
830, 780	−	−	−	−	−	Aromatic ring –C–H stretching and O–H bending out of plane	[49]

**Table 3:** Dimensions of NC, C–NC, and NC–PL.

Samples	Diameter (nm)	Length (nm)
RF	92.1 × 10^3^ ± 3.8	–
CL	7.6 × 10^3^ ± 2.3	–
NC	33.4 ± 11.4	224.8 ± 85.4
C–NC	44.3 ± 18.1	265.3 ± 64.1
NC–PL	53.6 ± 15.1	363.5 ± 103.6

Mean ± standard deviation values.

**Table 4:** Size and zeta potential of PL, NC, C–NC, and NC–PL.

Samples	DLS (nm)	Zeta Potential (mV)
PL	174.1 ± 15.3	48.7 ± 3.7
NC	112.1 ± 3.4	−64.7 ± 1.5
C–NC	439.0 ± 14.1	−39.7 ± 1.7
NC–PL	867.0 ± 19.8	38.0 ± 1.3

Mean ± standard deviation values.

## Data Availability

The original contributions presented in the study are included in the article/Supplementary Material, further inquiries can be directed to the corresponding author/s.

## Supplementary Materials

The following supporting information can be downloaded at: https://www.mdpi.com/article/10.3390/polym16131881/s1, Figure S1: Schematic of the reaction between NC, C–NC, and PL; Figure S2: Schematic of the chemical model of adsorption of FB1 by NC-PL.
