# Supplementary material for: Production of Nanocellulose from Sugarcane Bagasse and Development of Nanocellulose Conjugated with Polylysine for Fumonisin B1 Toxicity Absorption
Source: Polymers (Basel). 2024 Jul 1;16(13):1881. doi: 10.3390/polym16131881 (PMC11244476; doi:10.3390/polym16131881)
Supplement: Supplementary file 1 [file polymers-16-01881-s001.zip › polymers-3065522-supplementary.pdf]

Supplementary data

Production of Nanocellulose from Sugarcane Bagasse and Development of Nanocellulose Conjugated with Polylysine for Fumonisin B1 Toxicity Absorption

Parichat Thipchai , Korawan Sringarm, Winita Punyodom, Kittisak Jantanasakulwong , Sarinthip Thanakkasaranee, Rangsan Panyathip, Chaiwat Arjin and Pornchai Rachtanapun*

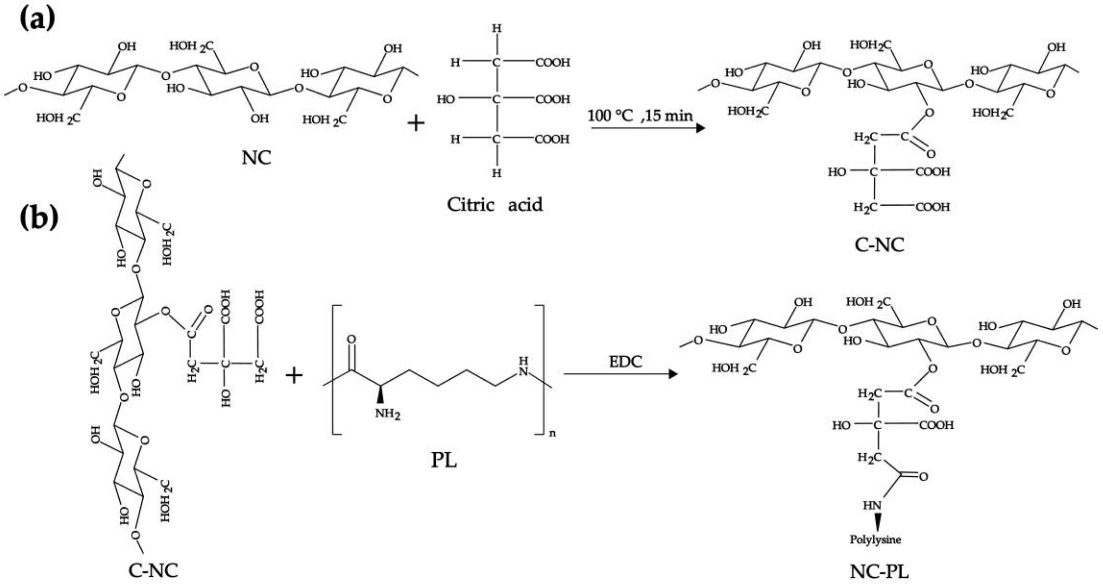

Figure S1. Schematic of (a) the reaction between citric acid and NC, (b) The reaction between C-NC and PL.

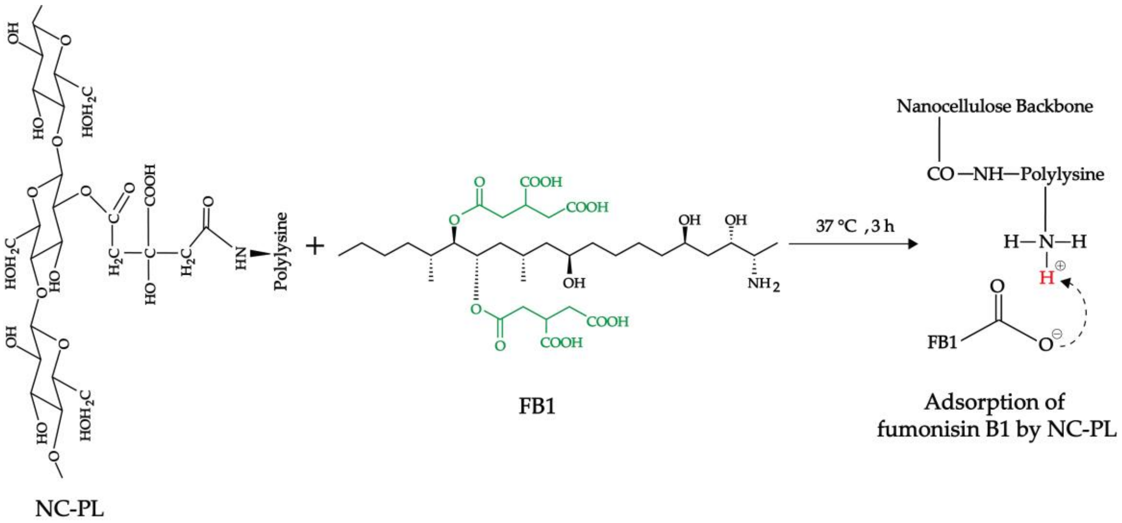

Figure S2. Schematic of the chemical model of adsorption of FB1 by NC-PL.
